# The L-type Ca^2+^ Channel Blocker Nifedipine Inhibits Mycelial Growth, Sporulation, and Virulence of *Phytophthora capsici*

**DOI:** 10.3389/fmicb.2016.01236

**Published:** 2016-08-04

**Authors:** Peiqing Liu, Jie Gong, Xueling Ding, Yue Jiang, Guoliang Chen, Benjin Li, Qiyong Weng, Qinghe Chen

**Affiliations:** Fujian Key Laboratory for Monitoring and Integrated Management of Crop Pests, Institute of Plant Protection, Fujian Academy of Agricultural SciencesFuzhou, China

**Keywords:** *Phytophthora capsici*, nifedipine, calcium rescue, virulence, H_2_O_2_

## Abstract

The oomycete vegetable pathogen *Phytophthora capsici* causes significant losses of important vegetable crops worldwide. Calcium and other plant nutrients have been used in disease management of oomycete pathogens. Calcium homeostasis and signaling is essential for numerous biological processes, and Ca^2+^ channel blockers prevent excessive Ca^2+^ influx into the fungal cell. However, it is not known whether voltage-gated Ca^2+^ channel blockers improve control over oomycete pathogens. In the present study, we compared the inhibitory effects of CaCl_2_ and the extracellular Ca^2+^ chelator EDTA on mycelial growth and found that calcium assimilation plays a key role in *P. capsici* mycelial growth. Next, we involved the voltage-gated Ca^2+^ channel blockers verapamil (VP) and nifedipine (NFD) to analyze the effect of Ca^2+^ channel blockers on mycelial growth and sporulation; the results suggested that NFD, but not VP, caused significant inhibition. Ion rescue in an NFD-induced inhibition assay suggested that NFD-induced inhibition is calcium-dependent. In addition, NFD increased *P. capsici* sensitivity to H_2_O_2_ in a calcium-dependent manner, and extracellular calcium rescued it. Furthermore, NFD inhibited the virulence and gene expression related to its pathogenicity. These results suggest that NFD inhibits mycelial growth, sporulation, and virulence of *P. capsici*.

## Introduction

Calcium acts as a second messenger and plays a direct role in controlling the expression patterns of its signaling systems in fungi. It is essential for numerous intrinsic metabolic processes including spore germination, hypha tip growth and branching, sporulation, hypha infection structure differentiation, circadian clocks, and responses to various environmental stresses (Liu et al., 2015a). However, improper regulation of Ca^2+^ in fungi can produce significant damages and even ultimately lead to cell death (Hu et al., 2013; Gonçalves et al., 2014; Liu et al., 2015b). Normally, calcium channels allow the passive flow of Ca^2+^ across cell membranes into the cytosol. Two major calcium uptake pathways have been identified in *Saccharomyces* and other fungi: the high-affinity (HACS) and low-affinity (LACS) calcium uptake systems (Martin et al., 2011; Wang et al., 2012; Harren and Tudzynski, 2013). The Cch1 and Mid1 Ca^2+^ channel complex constitutes the HACS (Cch1 functions as the pore, and Mid1 serves as a assistance) that mediates the specific influx of Ca^2+^ (Cavinder et al., 2011; Harren and Tudzynski, 2013). Mid1 and Cch1 have been identified in many filamentous fungi (Hallen and Trail, 2008; Yu et al., 2012), and deletion of Mid1 affects vegetative growth, cell wall synthesis, and virulence in *Claviceps purpurea* (Bormann and Tudzynski, 2009). In *Botrytis cinerea*, Cch1 and Mid1 are functionally required for vegetative growth under low-calcium conditions (Harren and Tudzynski, 2013). In *Gibberella zeae*, Mid1 affects the hypha growth, development processes, and even ascospore discharge significantly (Cavinder et al., 2011). In *Cryptococcus neoformans*, knock-out of Mid1 or Cch1 can caused significantly inhibition to oxidative stress (Vu et al., 2015).

In fact, voltage-gated Ca^2+^ channel blockers function in various modes. VP, one of the important L-type calcium channel blocker, is widely used in the medical treatment and served as a miracle drug in the treatment of angina pectoris and even hypertension. It partially inhibits the function of HACS, resulting in decreased calcium influx under normal growth conditions in *S. cerevisiae* and *Candida albicans* (Breeuwer et al., 1995; Yu et al., 2014). Nifedipine (NFD), used as a dihydropyridine derivative commonly, forms a stable complex with the L-type calcium receptors’ binding site, which is made up of six spatially separated amino acid residues while its conformation corresponds to the closed channel. NFD preferentially blocks Ca^2+^ channels of various cell types and prevents Ca^2+^ influx by reducing cytosolic Ca^2+^ concentrations (Nguemo et al., 2013). Diltiazem, a benzothiazepine-type calcium channel blocker, blocks L-type calcium channel by way of their high-affinity binding (Hockerman et al., 2000). The voltage-gated Ca^2+^ channel blockers can be used to treat the fungal pathogen *C*. *albicans* (Yu et al., 2014), and diltiazem and VP can block the opening of voltage-gated L-type Ca^2+^ channels and prevent a severe Ca^2+^ influx into the animal cells and *S. cerevisiae* (Teng et al., 2008). High concentrations of diltiazem also resulted in enhanced Ca^2+^ accumulation in *S. cerevisiae* cells (Binder et al., 2010). In addition, chelating extracellular calcium modulates cytosolic calcium, which severely reduces the expression of several calcium transport proteins and influences the normal functions of fungi (Juvvadi et al., 2015; Puigpinós et al., 2015). The reduction of intracellular calcium is responsible for the inhibition of reactive oxygen species (ROS)-generating enzymes and formation of free radicals by the mitochondria respiratory chain (Gordeeva et al., 2003; Kraus and Heitman, 2003). The Cch1-Mid1 regulated HACS contributes to a virulence change in *C. neoformans* by mitigating oxidative stress (Vu et al., 2015), and VP has an inhibitory effect on the oxidative stress response in *C*. *albicans* (Yu et al., 2014), confirming the relationship between calcium signaling and oxidative stress.

The oomycete vegetable pathogen *P. capsici* is a virulent, hemibiotrophic pathogen of vegetable crops, which inflicts significant losses of important vegetable crops worldwide (Jackson et al., 2012). Although it was first described almost 90 years ago, no direct and effective managements have been developed (Lamour et al., 2012). *P. capsici* has shown remarkable adaptation to fungicides and new hosts. Cinnamaldehyde (CA) is a major constituent of cinnamon essential oils and has been used as a food antimicrobial agent for its inhibiting of bacteria, yeast, and filamentous fungus, which involves membrane action, cell wall synthesis, and specific cellular processes (Wang et al., 2005; Shreaz et al., 2010). Previous studies also have shown that calcium eﬄux is involved in CA-induced inhibition of *P. capsici* zoospores (Hu et al., 2013). In addition, treatment with the voltage-gated calcium channel blocker verapamil (VP) can lead to a higher level of CA-induced Ca^2+^ eﬄux, suggesting that the Ca^2+^ channel may be a target for controlling pathogens. Furthermore, plant nutrients, especially Ca^2+^, can be applied to the disease management in *Phytophthora* spp. (Sugimoto et al., 2005), and more attention has focused on the suppressive effect of calcium on *Phytophthora* spp. (Sugimoto et al., 2010). In fact, CaCl_2_ and Ca(NO_3_)_2_ can dramatically suppress disease incidence caused by *P. sojae* in black soybean and white soybean and affect plant resistance. Moreover, 4–30 mM CaCl_2_ and Ca(NO_3_)_2_ can decrease the release of *P. sojae* zoospores (Sugimoto et al., 2005). Although voltage-gated Ca^2+^ channel blockers have been used widely, it is not known whether they could be used to control oomycete pathogens, especially *P. capsici.* In the present study, we investigated the effects of voltage-gated Ca^2+^ channel blockers on *P. capsici* mycelial growth, sporulation, and virulence.

## Materials and Methods

### *Phytophthora capsici* Strains and Culture Conditions

The *P. capsici* genome-sequenced virulence strain LT1534 was provided by Prof. Lamour (University of Tennessee, Knoxville, TN, USA), which has been used as a model strain by more and more scientists (Stam et al., 2013; Iribarren et al., 2015; Liu et al., 2016). Strain LT1534 was grown on 10% V8 juice agar medium at 25°C in the dark (Lamour et al., 2012). Radial growth was measured at day 5, when the colony of the strain LT1534 had almost completely covered the surface of the media in the dishes. Radial growth was assessed by measuring the distance from the edge of the inoculum plug to the advancing margin of the colony.

### Effect of CaCl_2_ and EDTA on Mycelial Growth of *P. capsici*

To test the inhibitory effect of CaCl_2_ and the extracellular Ca^2+^ chelator EDTA on *P. capsici* mycelial growth, strain LT1534 was grown on 10% V8 juice agar medium at 25°C, and then 1-week-old agar plugs (5 mm diameter) transferred onto the center of dish (10% V8 juice agar medium containing 0, 2.5, 5, 10, 25, and 50 mM CaCl_2_, and 0, 0.5, 1.0, 2.5, 5, and 10 mM EDTA). Radial growth was measured at day 5. Stock solutions of CaCl_2_ and EDTA were prepared as 1 M CaCl_2_ in H_2_O (Sigma–Aldrich) and 1 M EDTA in H_2_O (Sigma–Aldrich).

### Effect of VP and NFD on Mycelial Growth and Sporulation

To analyze the growth inhibitory effect of VP and NFD on *P. capsici* strain LT1534, 0, 10, 40, 80, 160, and 320 μg/mL VP and 0.1, 0.2, 0.5, 1, and 2 μM NFD were added to 10% V8 juice agar medium, and radial growth was measured at day 5. Stock solutions of VP and NFD were prepared as 320 mg/mL VP in H_2_O (Sigma–Aldrich) and 50 mM NFD in DMSO (Sigma–Aldrich).

To analyze zoosporangia density, the mycelia were washed three times with 30 mL of sterile distilled water and then an additional 20 mL of sterile distilled water was added to induce sporangia formation in the dark at 25°C for 24 h. The number of zoosporangia was counted and the mean of three duplications was used as the result of one replicate. Each experiment was repeated in triplicate wells at least three times.

To investigate whether calcium is associated with the inhibitory effect of NFD, strain LT1534 was grown on 10% V8 juice agar medium at 25°C, and then 1-week-old agar plugs (5 mm diameter) transferred onto the center of dish (10% V8 juice agar medium containing 0.5 μM NFD (Control), 0.5 μM NFD+20 mM CaCl_2_, 0.5 μM NFD+50 mM CaCl_2_, 0.5 μM NFD+20 mM KCl, and 0.5 μM NFD+20 mM NaCl). Radial growth was measured at day 5.

### Determination of Cytosolic Free Ca^2+^ Levels by Using the Probe Fluo-3-AM

*P. capsici* strain LT1534 was cultured for 2–3 days on the 10% V8 juice agar medium containing 0.5 μM NFD. Fluo-3-AM was prepared from a 1 mM stock solution in DMSO (Sigma) and added to the small pieces (1 cm × 1 cm) of *P. capsici* to a final concentration of 150 μM. The cultures were incubated at 37°C for 1 h for dye loading. Images of calcium green fluorescence were observed under a Nikon microscope by using a 450- to 490-nm excitation filter and a 520-nm barrier filter.

### Sensitivity Test to Oxidative Stress during NFD Treatment

To test the sensitivity of mycelial growth to oxidative stress, strain LT1534 was grown on 10% V8 juice agar medium at 25°C, and then H_2_O_2_ was added onto 10% V8 juice agar medium at final concentrations of 0, 1.25, 2.5, 5, and 10 mM. To investigate the effect of NFD on *P. capsici* mycelial growth under H_2_O_2_, radial growth was measured at 25°C for 5 days in V8 medium containing 0.5 μM NFD, 5 mM H_2_O_2_, 20 mM CaCl_2_, 0.5 μM NFD+1.25 mM H_2_O_2_, 0.5 μM NFD+2.5 mM H_2_O_2_, 0.5 μM NFD+5 mM H_2_O_2_, 0.5 μM NFD+10 mM H_2_O_2_, 0.5 μM NFD+10 mM H_2_O_2_ +20 mM CaCl_2_, and 0.5 μM NFD+10 mM H_2_O_2_ +50 mM CaCl_2_.

### Virulence Test

Zoospores were induced from 5-day-old sporangia by washing with sterile distilled water for 24 h at 25°C, and then harvested by centrifugation at 3000 × *g* for 5 min. The number of zoospores in 10 μL of zoospore suspension was counted using a blood cell counting chamber. Pepper cultivars (*Capsicum annuum* L. cv. yanshan01, CM334, and ECW) were collected from Yunnan province, China (Liu et al., 2015b) and grown in plastic pots containing vermiculite at 25°C for 4 days in the dark. The hypocotyls of etiolated seedlings were inoculated with 100 zoospores, and then etiolated seedlings were maintained in 80% humidity and darkness at 25°C. The pathogenicity results were investigated and photographs were taken 3 days post-inoculation (dpi).

### RNA Extraction and Quantitative Reverse Transcription Polymerase Chain Reaction (qRT-PCR)

Total RNA of mycelia was extracted using an RNA kit (Tiangen, China), and cDNA was generated according to the protocol of the PrimeScript RT reagent kit (TaKaRa). qRT-PCR assays were performed using the primers shown in **Table 1** and carried out in a BioRad CFX96 Real-Time PCR Detection instrument (Bio-Rad Laboratories) using standard PCR conditions. To confirm product specificity, we performed a melting curve analysis. Normally, a 20-μL reaction volume contained 2 μL of reverse transcription product, 10 μL of SYBR real-time PCR mix (2x), and 0.4 μL of each primer (10 μM). The *P. capsici* internal transcribed spacer (ITS) region was used as a constitutively expressed endogenous control, and the expression of each gene in **Table 1** was determined relative to the *P. capsici* ITS region using the ΔΔ*C*t method. qRT-PCR experiments were repeated in triplicate with independent RNA isolations.

**Table 1:** Sequences of primers used in the present study.

Gene	Primer sequence (5′–3′)
*P. capsici ITS region*	Forward: GTATAGCAGAGGTTTAGTGAA
	Reverse: GACGTTTTAGTTAGAGCACTG
*PcLAC2*	Forward: CTCATCAACTCAGTCACA
	Reverse: GGTTCTGCTTGGAATTAG
*PcPL16*	Forward: CCGACCTTGTCACTTATG
	Reverse: TGTTGTTGATTCCGAGAG

### Statistical Analysis

All measurements were conducted at least three times. Data were analyzed by one-way analysis of variance (ANOVA) using SPSS software version 19.0 (IBM) and mean comparison was conducted by a Dunnett’s test. Different letters above bars indicate statistical differences (^∗∗^*P* < 0.01 and ^∗^*P* < 0.05).

## Results

### Effect of Calcium on *P. capsici* Mycelial Growth

**FIGURE 1:**
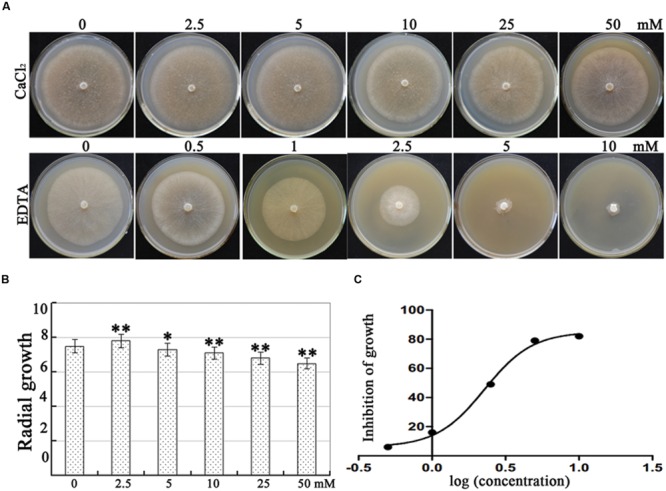
**Effect of CaCl_2_ and EDTA on mycelial growth of *Phytophthora capsici*. (A)** *P*. *capsici* cultures in Petri dishes illustrating the inhibition of mycelial radial growth with increasing CaCl_2_ and EDTA concentrations. The mycelial colonies were 5 days old and radial growth (mm) was assessed by measuring the distance from the edge of the inoculum plug to the advancing margin of the colony. **(B)** Statistical analysis of the inhibition of *P*. *capsici* mycelial growth at various CaCl_2_ concentrations. **(C)** Statistical analysis of the inhibition of *P*. *capsici* mycelial growth at various EDTA concentrations. The assays were repeated three times; *n* = 5 for each assay. EC50 is the concentration at which growth is inhibited by 50%. Trend-lines were fitted using a logarithmic function. Different letters above bars indicate statistical differences (^∗∗^*P* < 0.01 and ^∗^*P* < 0.05, according to Dunnett’s test).

Plant nutrients are often used in disease management, and the application of CaCl_2_ dramatically suppresses disease incidence caused by *P. sojae* in soybean under laboratory conditions and in field applications (Sugimoto et al., 2005). In the present study, we analyzed the effects of CaCl_2_ (0, 2.5, 5.0, 10, 25, and 50 mM) and the extracellular Ca^2+^ chelator EDTA (0, 0.5, 1.0, 2.5, 5.0, and 10 mM) on *P*. *capsici* virulence strain LT 1534 mycelial growth. In our results, 2.5 mM CaCl_2_ increased mycelial growth; however, growth was inhibited at 5 mM, reaching only 13% growth rate at 50 mM. In addition, EDTA reduced mycelial growth significantly and the half maximal effective concentration (EC_50_) value for EDTA-induced inhibition of mycelial growth was 2.5 mM (**Figure 1**). These results showed that calcium is essential, but higher concentrations are destructive, suggesting that *P. capsici* mycelial growth is regulated by calcium assimilation significantly.

### Nfd, But Not Vp, Inhibits *P. capsici* Mycelial Growth and Sporulation Significantly

**FIGURE 2:**
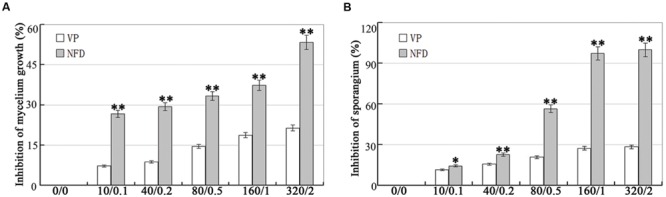
**Effects of verapamil (VP) and nifedipine (NFD) on mycelial growth and sporulation of *P. capsici*. (A)** Analysis of the inhibition of *P*. *capsici* mycelium growth at various VP and NFD concentrations. The mycelial colonies were 5 days old and radial growth (mm) was assessed by measuring the distance from the edge of the inoculum plug to the advancing margin of the colony. **(B)** Analysis of the inhibition of *P*. *capsici* sporulation at various VP and NFD concentrations. The assays were repeated three times; *n* = 5 for each assay. Different letters above bars indicate statistical differences (^∗∗^*P* < 0.01 and ^∗^*P* < 0.05, according to Dunnett’s test).

**FIGURE 3:**
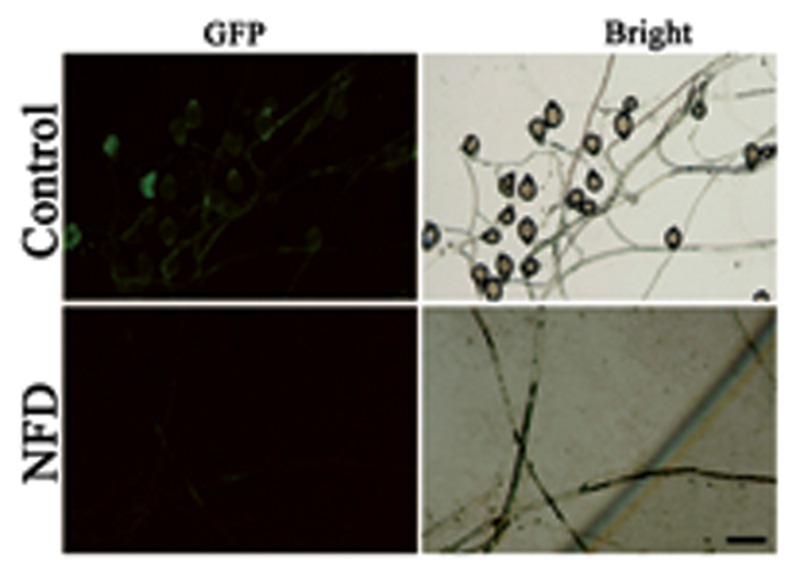
**Cytosolic Ca^2+^ images of *P. capsici* observed by light microscopy and fluorescence microscopy when NFD was added.** Fluo-3-AM (150 μM) was added to the small pieces (1 cm × 1 cm) of *P. capsici*. The intensities of green fluorescence represent the relative amounts of free cytosolic Ca^2+^. Bar = 20 μm.

Disruption of the genes encoding calcium channels results in abnormal calcium uptake for homeostasis and signaling, and impacts vegetative growth, polarity, cell wall integrity, and virulence (Bormann and Tudzynski, 2009; Wang et al., 2012). We evaluated the inhibitory effect of VP and NFD on mycelial growth and sporulation in *P. capsici*. As shown in **Figure 2**, mycelial growth and sporulation were inhibited by VP and NFD compared with the non-treated control. The highest dose of NFD (2 μM) inhibited mycelial growth and sporulation by 53 and 100%, but the highest dose of VP (320 μg/mL) only inhibited by 21 and 28%, suggesting that NFD inhibits mycelial growth and sporulation of *P. capsici* significantly. In addition, NFD led to concentration-dependent inhibition of *P. capsici* mycelial growth and sporulation, which peaked at 2 μM. Furthermore, NFD showed 30–35% greater inhibition of mycelial growth and 55–60% greater inhibition of sporulation. The above results suggest that NFD, but not VP, inhibits *P. capsici* mycelial growth and sporulation significantly. Furthermore, we also evaluated the inhibitory effect of NFD on the intensity of fluorescence emission representing the relative amounts of free intracellular Ca^2+^. As shown in **Figure 3**, strong green fluorescence was observed in the control. On the contrast, the fluorescence of NFD treated *P. capsici* decreased notably. These results suggest that NFD regulates the content of cytosolic free Ca^2+^ levels.

### Rescue of NFD-Inhibited Mycelial Growth by Extracellular Calcium

**FIGURE 4:**
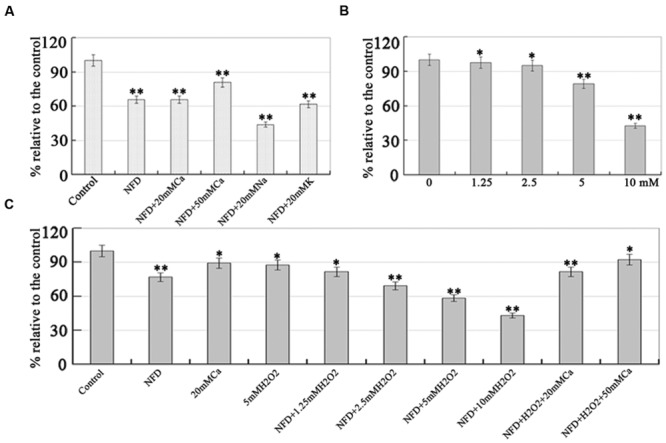
**Effects of CaCl_2_ and H_2_O_2_ on NFD-induced growth inhibition. (A)** Extracellular calcium rescued NFD-inhibited mycelial growth. Mycelial was grown on 10% V8 juice agar medium containing 0.5 μM NFD (Control), 0.5 μM NFD+20 mM CaCl_2_, 0.5 μM NFD+50 mM CaCl_2_, 0.5 μM NFD+20 mM NaCl, and 0.5 μM NFD+20 mM KCl. **(B)** Effect of H_2_O_2_ on *P. capsici* mycelial growth. Mycelial was grown on 10% V8 juice agar medium containing 0, 1.25, 2.5, 5, and 10 mM H_2_O_2_. **(C)** Effect of NFD on mycelial growth of *P. capsici* under various concentrations of H_2_O_2_. Mycelial was grown on 10% V8 juice agar medium containing 0.5 μM NFD, 20 mM CaCl_2_, 5 mM H_2_O_2_, 0.5 μM NFD+1.25 mM H_2_O_2_, 0.5 μM NFD+2.5 mM H_2_O_2_, 0.5 μM NFD+5 mM H_2_O_2_, 0.5 μM NFD+10 mM H_2_O_2_, 0.5 μM NFD+10 mM H_2_O_2_+20 mM CaCl_2_, and 0.5 μM NFD+10 mM H_2_O_2_+50 mM CaCl_2_. The mycelial colonies were 5 days old and radial growth (mm) was assessed by measuring the distance from the edge of the inoculum plug to the advancing margin of the colony. The assays were repeated three times; *n* = 5 for each assay. Different letters above bars indicate statistical differences (^∗∗^*P* < 0.01 and ^∗^*P* < 0.05, according to Dunnett’s test).

Next, we investigated whether the inhibitory effect of NFD could be rescued by extracellular calcium levels. As shown in **Figure 4A**, NFD treated alone inhibited mycelial growth by 33.7% compared with the non-treated control, and NFD+20 mM CaCl_2_ did not rescue NFD inhibited mycelial growth. However, the inhibition of mycelial growth in NFD+50 mM CaCl_2_ was 19%, suggesting that 50 mM CaCl_2_ can restrain NFD inhibited mycelial growth and rescue by 14.7%. To exclude non-specific (such as osmotic) effects of CaCl_2_ due to its high concentrations, other salts (e.g., NaCl and KCl) was employed to investigate the effect of NFD-induced reduction of mycelial growth. Our results showed that 20 mM NaCl and KCl do not increase cell growth. These results suggest that NFD-induced inhibition of mycelial growth is calcium-dependent.

### NFD Increased *P. capsici* Sensitivity to H_2_O_2_ in a Calcium-Dependent Manner

Adaptation of pathogens to plant-derived ROS is important for their successful infection (Ermak and Davies, 2002; Sheng et al., 2015). In the present study, 0, 1.25, 2.5, 5, and 10 mM H_2_O_2_ was used to investigate the effect of oxidative stress on *P. capsici* mycelial growth. As shown in **Figure 4B**, the mycelial growth was significantly inhibited by 55–60%, when treated with 10 mM H_2_O_2_. The inhibitory effect of H_2_O_2_ on *P. capsici* mycelial growth was concentration dependent, suggesting that *P. capsici* is sensitive to oxidative stress in an H_2_O_2_ concentration-dependent manner. Furthermore, the effect of NFD on *P. capsici* oxidative stress and whether 0.5 μM NFD influenced the sensitivity of *P. capsici* to H_2_O_2_ were analyzed. As shown in **Figure 4C**, the inhibition of mycelial growth by NFD or 5 mM H_2_O_2_ treatment were 23.1 and 12.3% compared with the non-treated control, but NFD+5 mM H_2_O_2_ treatment inhibited mycelial growth by 41.5%. Furthermore, NFD+10 mM H_2_O_2_ treatment inhibited mycelial growth by 57%, suggesting that NFD increased the sensitivity of *P. capsici* to oxidative stress, which is dependent on the concentration of H_2_O_2_. However, NFD+H_2_O_2_ +50 mM CaCl_2_ treatment restrained NFD+10 mM H_2_O_2_ inhibited mycelial growth and rescue by 49.2%, suggesting that 50 mM CaCl_2_ can rescue the inhibitory effect of NFD+10 mM H_2_O_2_. Therefore, NFD increased *P. capsici* sensitivity to H_2_O_2_, and extracellular calcium rescued it.

### NFD Inhibition of *P. capsici* Virulence and Expression of Genes Involved in Pathogenicity

**FIGURE 5:**
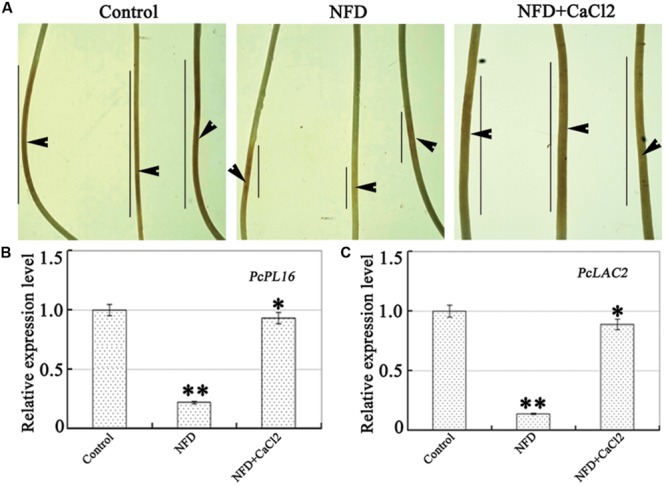
**Effect of NFD on *P. capsici* virulence and expression of genes involved in pathogenicity. (A)** Pathogenicity assays were performed on etiolated pepper seedlings. Etiolated pepper seedlings (*C*. *annuum* L. cv. yanshan01, CM334, and ECW) were inoculated with freshly prepared zoospores (∼100 in 10 mL). Images were taken after 48 h, and the experiments were repeated at least three times. Arrows indicate the inoculation site and lines indicate the lesion size. **(B,C)** Relative transcription levels of defense-related genes *PcPL16* and *PcLAC2* in NFD-treated mycelia. Transcription of the *P. capsici* ITS region in mycelia was used as a reference, which was set to a value of 1. Error bars indicate the standard error. The experiments were repeated three times, together with at least three independent repetitions of the biological experiments. Values are means ± SE (*n* = 6). Different letters above bars indicate statistical differences (^∗∗^*P* < 0.01 and ^∗^*P* < 0.05, according to Dunnett’s test).

Zoospore suspensions were collected from strain LT1534 grown on 10% V8 juice agar medium containing NFD and inoculation assays were performed on etiolated *C. annuum* L. seedlings. As shown in **Figure 5A**, the hypocotyls of the etiolated seedlings inoculated with strain LT1534 zoospores showed typical disease symptoms and water-soaked lesions at 3 dpi. In contrast, NFD-treated strain LT1534 produced almost no lesions or very small lesions which did not expand beyond the inoculation site (**Figure 5A**). To determine whether the pathogenicity defect was associated with the expression of pathogenicity-related genes during infection by *P. capsici*, we analyzed the relative expression ratios of the pectate lyase *PcPL16* and laccase *PcLAC2* genes. As shown in **Figures 5B,C**, the expression levels of *PcPL16* and *PcLAC2* were markedly higher in mycelia grown on V8 medium. In contrast, the expression levels of *PcPL16* and *PcLAC2* in the NFD-treated mycelia were significantly lower. Furthermore, the addition of calcium rescued the virulence and expression of *PcPL16* and *PcLAC2* to the levels observed in V8 medium. These results suggest that NFD inhibited the virulence and expression of pathogenicity-related genes in *P. capsici*.

## Discussion

Calcium eﬄux is involved in the fungicide CA-induced inhibition of *P. capsici* (Hu et al., 2013). The antifungal protein PAF from *Penicillium chrysogenum* has been used to control disease by increasing cytosolic free Ca^2+^, which is involved in hyphal tip growth, hyphal branching, sporulation, spore germination, different infection structure formation, circadian clocks, and responses to environment stimuli including osmotic stress, heat shock, mechanical stimulations, and oxidative stresses. Calcium channels allow the passive flow of Ca^2+^ across cell membranes into the cytosol. Voltage-gated Ca^2+^ channel blockers can inhibit the growth of fungal pathogens (Binder et al., 2010), but little is known about the effect of Ca^2+^ homeostasis on mycelial growth, sporulation, and virulence of oomycetes. NFD is the prototype calcium channel blocker of the dihydropyridine class, and the half-life of capsule and tablet are 2 and 11 h for the management of hypertension and angina pectoris (Toal, 2004). Previous studies have shown that NFD can potentiate cardiopulmonary baroreflex control of sympathetic nerve activity (Ferguson and Hayes, 1989), inhibit contractions in the body of the human esophagus (Richter et al., 1985) and decrease lymphocyte blastogenesis, IL2 production and NK activity in healthy humans (Morgano et al., 1990). In the present study, we first explored the effects of two well-known calcium channel blockers, VP and NFD, on mycelial growth and sporulation. As shown in **Figure 2**, NFD, but not VP, inhibited mycelial growth and sporulation of *P. capsici* strain LT1534 significantly. Meanwhile, we also found that 2 μM NFD inhibited mycelial growth of Fujian and Jiangsu *P. capsici* strains significantly (**Supplementary Figure S1**), suggesting that NFD can inhibit mycelial growth of *P. capsici*. Lange and Peiter (2016) have shown that NFD drastically reduced colony growth in the filamentous fungal pathogen *Colletotrichum graminicola*, as observed before in *Fusarium graminearum*, and the affected growth to a much larger extent than external Ca^2+^ chelation; meanwhile, Scherp et al. (2001) also have shown that NFD is capable of stimulating the callose deposition in cells undergoing cytokinesis in *Riella helicophylla* and *Arabidopsis thaliana*, suggesting that NFD can be used in the crop protection. Extracellular calcium rescued NFD-inhibited mycelial growth (**Figure 4A**), suggesting that NFD-regulated calcium uptake is significantly beneficial for hyphal growth in *P. capsici*. In addition, NFD increased *P. capsici* sensitivity to H_2_O_2_ in a calcium-dependent manner (**Figure 4C**). Furthermore, oxidative stress alters calcium signaling, and calcium homeostasis and signaling is linked to pathogenesis (Hallen and Trail, 2008; Liu et al., 2015a).

The Ca^2+^ influx channels Cch1 and Mid1 in *S. cerevisiae* allow the passive flow of Ca^2+^ across cell membranes into the cytosol (Harren and Tudzynski, 2013), and the Cch1-Mid1 complex in *Aspergillus fumigatus* mediates the specific influx of Ca^2+^; calcium uptake impacts conidiation, vegetative growth, and polarity (Jiang et al., 2014). In the present study, we analyzed the effects of VP and NFD on mycelial growth and sporulation of *P. capsici*, and showed that VP functions in a mechanism that differs from that of NFD. In fact, NFD and VP use different binding sites in the cell wall (Nakayama and Kanaoka, 1996), and NFD and VP are members of the chemically unrelated classes of L-type blockers, dihydropyridines and phenylalkylamines, respectively. In addition, different inhibitory effects of three L-type calcium blockers (diltiazem, VP, and NFD) on ADP- and collagen-induced platelet aggregation of human and rabbit platelets have been reported (Toque et al., 2008). In fact, the bioavailabilities of diltiazem, NFD, and VP differ with ranges of 40–50%, 40–50%, and 10–30%, respectively (Echizen and Eichelbaum, 1986).

Previous studies have suggested that HACS is involved in the oxidative stress response, and the calcium channel blocker VP inhibits the oxidative stress response in *C. albicans* (Yu et al., 2014). In addition, deletion of three HACS regulator-encoding genes *Cch1, Mid1*, and *Ecm7* results in increased sensitivity to oxidative stress and decreased expression of several oxidative stress response genes (Ding et al., 2013). In the present study, NFD increased the sensitivity of *P. capsici* to H_2_O_2_ in a calcium-dependent manner, suggesting that *P. capsici* treated with H_2_O_2_ and NFD encounters more severe oxidative stress than with H_2_O_2_ treatment alone. However, 50 mM extracellular calcium rescued NFD-reduced mycelial growth under oxidative stress, suggesting that NFD inhibits mycelial growth under oxidative stress by disrupting calcium fluctuation.

In the present study, NFD-treated *P. capsici* produced very small lesions which showed no expansion beyond the inoculation site; in contrast, treatment with NFD and CaCl_2_ showed typical disease symptoms (**Figure 5A**). In fungi, the changed pathogenicity may be due to infection-related enzymes and effector-related protein secretion. During infection, diverse cell wall-degrading enzymes (e.g., pectinase) can be produced on the infection sites by *Phytophthora* spp. Pectinases degrade pectin, which is a major component of the primary cell wall and middle lamella of plants. Recent studies of the biological function of fungal laccases suggest that this enzyme plays an important role in fungal morphogenesis and fungal virulence (Li et al., 2013). In *P. capsici*, pectate lyase and laccase activities are important for successful infection during plant–pathogen interactions (Feng and Li, 2014; Fu et al., 2015). Laccases, which served as blue copper oxidases, catalyze the one-electron oxidation (e.g., aromatic amines and phenolics) and other electron-rich substrates; there also has a reduction of O_2_ to H_2_O concomitantly. In the present study, the expression levels of laccase *PcLAC2-* and pectate lyase *PcPL16*-encoding genes were reduced significantly by 35–50% in NFD- and H_2_O_2_-treated *P. capsici*. In fact, *Bacillus subtilis* pectate lyase is in a complex with calcium (Pickersgill et al., 1994) and *Rhizoctonia solani* laccase activity is induced by CaCl_2_ (Crowe and Olsson, 2001). The promoter regions of laccase genes have several putative *cis*-acting elements such as xenobiotic-responsive, metal-responsive, and stress-responsive elements. In addition, effector proteins function not only as toxins to induce plant cell death, but also to enable pathogens to suppress or evade plant defense responses. Necrosis-inducing NLP proteins have been reported to contribute strong virulence during infection by *P. capsici* (Feng et al., 2014). In our study, NFD treatment alone had no obvious effect on the expression of *PcLAC2* and *PcPL16*. Successful rescue by CaCl_2_ suggested that NFD-inhibited Ca^2+^ absorption is important for virulence. Therefore, plants can respond to pathogenic fungi or oomycete infection by rapidly producing ROS using membrane-bound NADPH oxidases or secreted peroxidases and amine oxidases, as part of the general pathogen-associated molecular pattern (PAMP)-triggered immunity or more specific effector-triggered immunity responses (Latijnhouwers et al., 2003).

In the present study, our results showed that the calcium channel blocker NFD has an inhibitory effect on *P. capsici* calcium fluctuation under oxidative stress and impacts the oxidative stress response, confirming a connection between calcium signaling and the oxidative stress response in this pathogen. Interestingly, Scherp et al. (2001) have shown that NFD is capable of stimulating the callose deposition in cells undergoing cytokinesis in *Riella helicophylla* and *Arabidopsis thaliana*, and Larkindale and Knight (2002) also have shown that NFD increases heat stress in *Arabidopsis thaliana*, suggesting that there has no phytotoxicity in NFD application. Therefore, calcium channels may be potential targets for therapy to enhance the efficacy of oxidative stress against *P. capsici*-related infections and NFD can be used to the crop protection safely.

## Author Contributions

PL, QW, and QC designed the study. JG, XD, YJ, BL, and GC performed the experiments. All authors analyzed the data. PL, QW, and QC wrote the article. All authors contributed to the research and manuscript and read and approved the final version of the manuscript. All authors agree to be accountable for all aspects of the work.

## Conflict of Interest Statement

The authors declare that the research was conducted in the absence of any commercial or financial relationships that could be construed as a potential conflict of interest.

## Supplementary Material

FIGURE S1 **Effects of nifedipine (NFD) on mycelial growth of Fujian and Jiangsu *P. capsici* strains.** Analysis of the inhibition of *P*. *capsici* strains PCZZ-10 and PCNJ9 mycelium growth at 2 μM NFD. The mycelial colonies were 5 days old and radial growth (mm) was assessed by measuring the distance from the edge of the inoculum plug to the advancing margin of the colony.
