# Supplementary figures and images for: The L-type Ca2+ Channel Blocker Nifedipine Inhibits Mycelial Growth, Sporulation, and Virulence of Phytophthora capsici
Source: Front Microbiol. 2016 Aug 4;7:1236. doi: 10.3389/fmicb.2016.01236 (PMC4972815; doi:10.3389/fmicb.2016.01236)

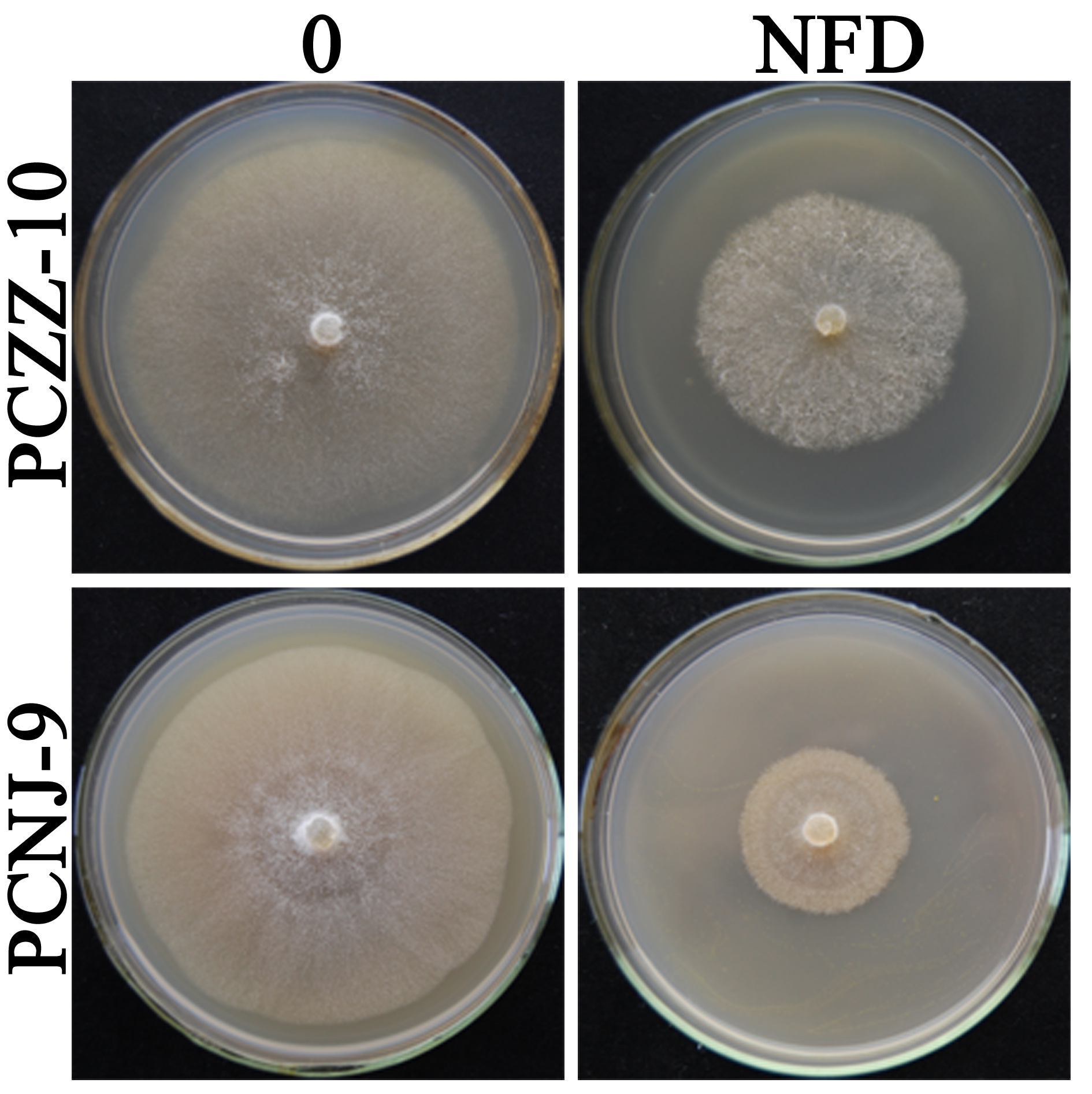

Supplement: FIGURE S1 — Effects of nifedipine (NFD) on mycelial growth of Fujian and Jiangsu P. capsici strains. Analysis of the inhibition of P. capsici strains PCZZ-10 and PCNJ9 mycelium growth at 2 μM NFD. The mycelial colonies were 5 days old and radial growth (mm) was assessed by measuring the distance from the edge of the inoculum plug to the advancing margin of the colony. [file Image_1.JPEG]
